# Sepsid *even-skipped* Enhancers Are Functionally Conserved in *Drosophila* Despite Lack of Sequence Conservation

**DOI:** 10.1371/journal.pgen.1000106

**Published:** 2008-06-27

**Authors:** Emily E. Hare, Brant K. Peterson, Venky N. Iyer, Rudolf Meier, Michael B. Eisen

**Affiliations:** 1 Department of Molecular and Cell Biology, University of California Berkeley, Berkeley, California, United States of America; 2 Center for Integrative Genomics, University of California Berkeley, Berkeley, California, United States of America; 3 Department of Biological Sciences, National University of Singapore, Singapore; 4 Genomics Division, Ernest Orlando Lawrence Berkeley National Laboratory, Berkeley, California, United States of America; 5 California Institute for Quantitative Biosciences, Berkeley, California, United States of America; Harvard Medical School, Howard Hughes Medical Institute, United States of America

## Abstract

The gene expression pattern specified by an animal regulatory sequence is generally viewed as arising from the particular arrangement of transcription factor binding sites it contains. However, we demonstrate here that regulatory sequences whose binding sites have been almost completely rearranged can still produce identical outputs. We sequenced the *even*-*skipped* locus from six species of scavenger flies (Sepsidae) that are highly diverged from the model species *Drosophila melanogaster*, but share its basic patterns of developmental gene expression. Although there is little sequence similarity between the sepsid *eve* enhancers and their well-characterized *D. melanogaster* counterparts, the sepsid and *Drosophila* enhancers drive nearly identical expression patterns in transgenic *D. melanogaster* embryos. We conclude that the molecular machinery that connects regulatory sequences to the transcription apparatus is more flexible than previously appreciated. In exploring this diverse collection of sequences to identify the shared features that account for their similar functions, we found a small number of short (20–30 bp) sequences nearly perfectly conserved among the species. These highly conserved sequences are strongly enriched for pairs of overlapping or adjacent binding sites. Together, these observations suggest that the local arrangement of binding sites relative to each other is more important than their overall arrangement into larger units of *cis*-regulatory function.

## Author Summary

The transformation of a fertilized egg into a complex, multicellular organism is a carefully choreographed process in which thousands of genes are turned on and off in specific spatial and temporal patterns that confer distinct physical properties and behaviors on emerging cells and tissues. To understand how an organism's genome specifies its form and function, it is therefore necessary to understand how patterns of gene expression are encoded in DNA. Decades of analysis of the fruit fly *Drosophila melanogaster* have identified numerous regulatory sequences, but have not fully illuminated how they work. Here we harness the record of natural selection to probe the function of these sequences. We identified regulatory sequences from scavenger fly species that diverged from *Drosophila* over 100 million years ago. While these regulatory sequences are almost completely different from their *Drosophila* counterparts, they drive identical expression patterns in *Drosophila* embryos, demonstrating extreme flexibility in the molecular machines that interpret regulatory DNA. Yet, the identical outputs produced by these sequences mean they must have something in common, and we describe one shared feature of regulatory sequence organization and function that has emerged from these comparisons. Our approach can be generalized to any regulatory system and species, and we believe that a growing collection of regulatory sequences with dissimilar sequences but similar outputs will reveal the molecular logic of gene regulation.

## Introduction

Recent studies revealing how the gain, loss and repositioning of transcription factor binding sites within regulatory sequences can alter gene expression with observable phenotypic consequences [1] have focused efforts to understand the molecular basis for organismal diversity on the evolution of regulatory DNA. However, a growing body of work has demonstrated that alterations of binding-site composition and organization often leave regulatory sequence function unchanged [2]–[9].

The potential for significant changes in regulatory sequences to have no functional consequences complicates efforts to identify sequence changes that are likely to affect gene expression and phenotype. But precisely because many of these changes do not affect regulatory output, they provide a powerful opportunity to understand how the arrangement of transcription factor binding sites in a regulatory sequence determines its output. We believe that identifying divergent enhancers that drive similar patterns of expression, and distilling the common principles that unite them, will allow us to decipher the molecular logic of gene regulation.

**Figure 1:**
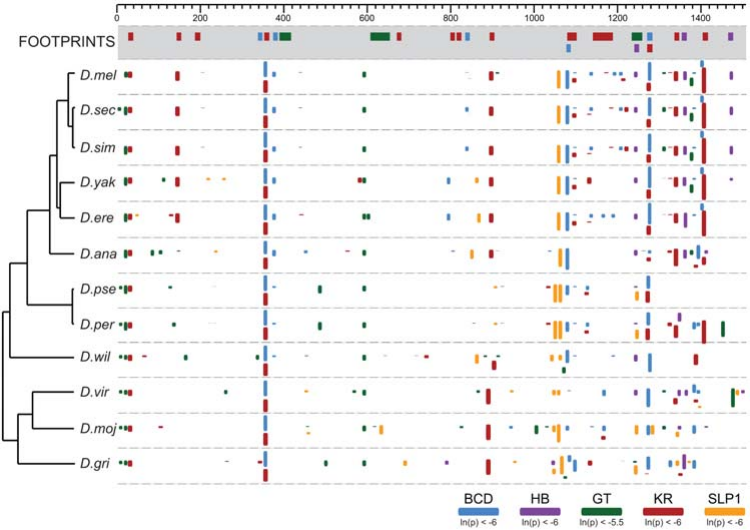
Binding site conservation and turnover in *Drosophila even-skipped* stripe 2 enhancer. Predicted binding sites for the five factors known to regulate expression from the *eve* stripe 2 enhancer in the twelve sequenced *Drosophila* species [56]. Sites were predicted independently in each species using PATSER [61] and mapped onto an MLAGAN [65] multiple alignment of the *eve* stripe 2 enhancer sequences. The height of the box representing each binding site is scaled by its PATSER p-value (taller boxes represent sites with higher predicted affinities). The top panel (grey shading) shows the positions of biochemically-verified (in vitro footprinting) binding sites [27]. The indicated coordinates are for the multiple-alignment, which is longer than individual enhancers due to the high frequency of alignment gaps.

We began to explore the effectiveness of this approach with the extensively studied regulatory systems of the early *D. melanogaster* embryo [10], using the recently sequenced genomes of 12 *Drosophila* species to document the evolutionary fate of transcription factor binding sites in early embryonic enhancers (Peterson, Hare, Iyer, Eisen, unpublished). A consistent pattern emerged: while binding site turnover is common, a large fraction of the binding sites in most enhancers are conserved across the genus (see Figure 1).

The extent to which variation in enhancers from sequenced *Drosophila* species represented all of the possible variation in these sequences was unclear. Perhaps the conserved sites were an imperturbable core essential for each enhancer's function. Or, perhaps, there had simply not been enough time since the divergence of the genus for mutation to have generated alternative configurations that would produce identical expression patterns. To resolve this ambiguity it was necessary to reconstruct binding site turnover events that occurred over longer evolutionary timescales by comparing *Drosophila* enhancers to their counterparts in species from outside the genus. The appropriate species for such comparisons would share basic patterning mechanisms with *Drosophila* species, but be sufficiently diverged from *Drosophila* to provide significant additional data on the constraints on binding site turnover. Ideally, these species would be amenable to experimental analysis and have fully sequenced genomes.

Unfortunately, the closest available genome sequences were from several very distantly related mosquito species [11], whose most recent common ancestor with *Drosophila* lived approximately 220 million years ago. These sequences were unlikely to be informative because of several important differences between early-embryonic patterning in *Drosophila* and mosquitoes. Mosquitoes, for example, lack the primary anterior morphogen in *Drosophila*, the modified *Hox* gene Bicoid, which is found only in higher cyclorrhaphan Diptera (the “true flies”) [12].

With essentially no information on non-coding sequences and regulatory networks from flies outside the Drosophilidae, we reasoned that other groups within the Acalyptratae, the speciose 100 million year-old division of Diptera that includes *Drosophila*, represented the best compromise between our aims to maximize sequence divergence and minimize regulatory network divergence.

We selected three families, Sepsidae, Diopsidae and Tephritidae, that span acalyptrate diversity, have well-characterized phylogenies, and contain multiple species whose specimens could be readily obtained. In this paper we present results on gene regulation in sepsids, which, due to their small genomes, were the most amenable to genome analysis.

Specifically, we report the sequence and experimental characterization of the *even-skipped* locus from six sepsid species. The particular species were selected to include the major sepsid lineages, and, in several cases, because of the amenability of the species for embryological study. We chose to characterize multiple sepsid species to facilitate the identification of sepsid enhancers by intra-family comparisons [13],[14] and to enable comparisons of enhancer evolution between sepsids and drosophilids.

## Results

### Sequencing Sepsid *eve* Loci

The six sepsid species we selected for this study, *Sepsis punctum*, *Sepsis cynipsea*, *Dicranosepsis sp.*, *Themira superba*, *Themira putris* and *Themira minor*, have genome sizes that range from 134 Mb to 285 Mb (Table 1). We generated a whole-genome fosmid library for each species, identified *eve*-containing clones by hybridization with a species-specific *eve* probe generated by degenerate PCR, and shotgun sequenced the clones to an average 13× coverage (Table S1). We annotated the assembled sequences (Table S2) to identify all protein-coding genes with homologs in *D. melanogaster* (Figure S1).

**Table 1:** Genome and *eve* locus size in sepsids.

Species	Genome size (Mb)	*eve* locus size (Kb)
*Themira putris*	265	20.9
*Themira minor*	165	20.6
*Themira superba*	134	20.4
*Dicranosepsis sp.*	241	28.3
*Sepsis cynipsea*	215	23.9
*Sepsis punctum*	285	24.1

**Figure 2:**
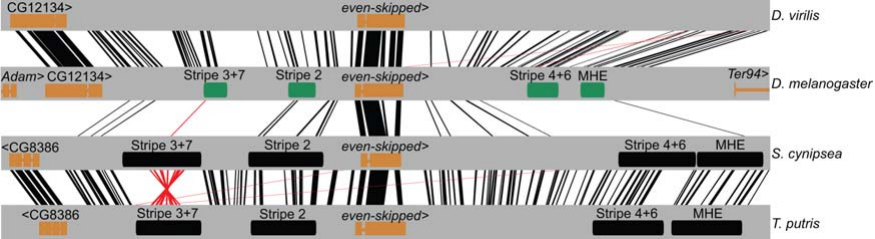
Conservation within and between sepsids and *Drosophila*. The *eve* locus (20 kb flanking the *eve* protein-coding gene) is shown for two *Drosophila* (*D. melanogaster* and *D. virilis*) and two sepsid species (*S. cynipsea* and *T. putris*), centered on the *eve* homeodomain. Black lines represent significant BLASTZ [15] hits on the plus strand, red lines on the minus strand (BLASTZ parameters: K = 1800, with chaining). Verified *D. melanogaster* enhancers are shown in green and predicted sepsid enhancers in black. Note that only a subset of *D. melanogaster eve* enhancers are shown here; all BLASTZ matches between *D. melanogaster* and *S. cynipsea* fall within known enhancers with the exception of those falling within 500 bp of the transcription start site in *D. melanogaster*.

All of the sequenced clones contained clear *eve* orthologs, and the organization of the *eve* locus is very similar in sepsids and drosophilids (Figure 2). The sepsid loci are slightly larger (Table 1), consistent with their overall larger genome sizes. The genes flanking *eve*, however, are different between the families.

### The Evolutionary Relationship of Sepsid and Drosophilid Species

**Figure 3:**
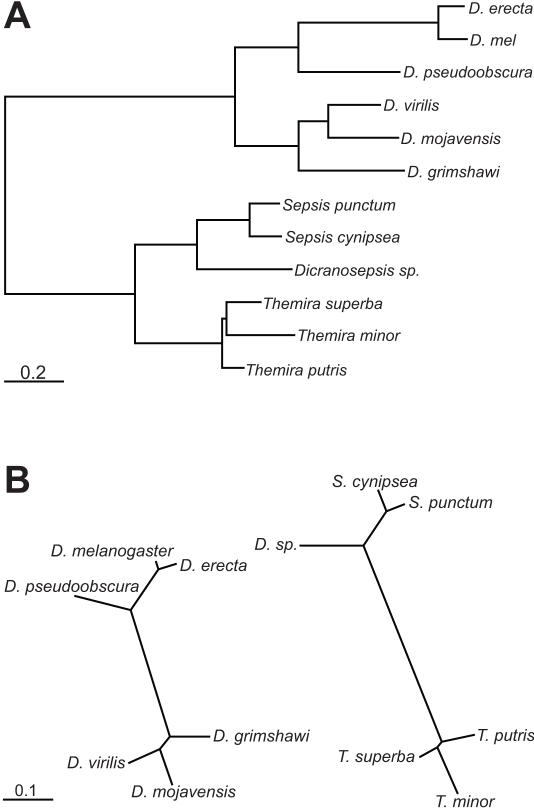
Coding and non-coding trees of sepsids and *Drosophila.* (A) Maximum likelihood tree of protein-coding genes inferred from seven genes using CODEML module of PAML [59]. Branch lengths are in substitutions per codon using the [F3×4] model. (B) Maximum likelihood non-coding trees of six *Drosophila* and six sepsids computed using the BASEML module of PAML [59]. Branch lengths are in substitutions per site using the HKY model.

A maximum likelihood tree calculated using seven protein-coding gene sequences in all six sepsids and a subset of *Drosophila* species demonstrates that the sepsid species are about twice as diverged from *D. melanogaster* than *D. melanogaster* is from the most distantly related *Drosophila* species (Figure 3A).

### Minimal Non-Coding Sequence Similarity between Sepsids and Drosophilids

Examination of the *eve* locus from sequenced *Drosophila* species shows that there is readily detectable non-coding sequence conservation spanning the entire locus, even between the most distantly related species (Figure 2). The average pairwise noncoding match score (a BLASTZ [15] based measure of sequence similarity; see Materials and Methods) between *D. melanogaster* and members of the virilis-repleta clade is 20% (Table S3). We observe a similar pattern in the sepsid *eve* loci. The average pairwise noncoding match score between *S. cynipsea* and *Themira* species is 17% (Table S3). However, there is minimal non-coding sequence conservation between families outside of a few small (approximately 20–30 bp) blocks of extremely high conservation scattered across the locus (Figure 2). The average pairwise noncoding match score between *D. melanogaster* and the sepsids is 4% (Table S3). Maximum likelihood non-coding trees from the *eve* locus in sepsids and *Drosophila* reveal that the two families span roughly the same amount of non-coding divergence (Figure 3B).

### Characterization of the *Trans*-Regulatory Network in the Sepsid *Themira minor*

**Figure 4:**
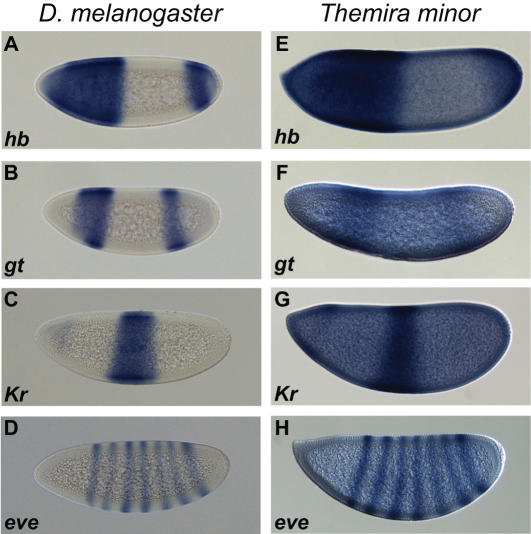
Expression of *eve* and its upstream transcriptional regulators is conserved between *Drosophila melanogaster* and the sepsid *Themira minor*. Expression patterns were visualized by *in situ* hybridization with species-specific digoxigenin-labeled antisense RNA probes. The gap transcription factors *hb*, *gt* and *Kr* are expressed in similar domains during stage 5 in *D. melanogaster* (A–C) and *T. minor* (E–G). *eve* is expressed in seven transverse stripes during cellularization in both species (D, H). Embryos are oriented with anterior to the left and dorsal up.

We established a colony of the *T. minor* from adults captured in Sacramento, CA, and developed protocols to recover and fix *T. minor* embryos. The overall morphology and pattern of embryonic development is very similar in sepsids and *Drosophila* (Figure S2). As expected from studies of other dipterans, *T. minor eve* is expressed in a characteristic set of seven stripes in blastoderm embryos (Figure 4D,H).

We were additionally interested in comparing the *trans-*regulatory network of this sepsid to that of drosophilids. In *D. melanogaster*, *eve* expression in the blastoderm is regulated by the transcription factors Bicoid (BCD), Caudal (CAD), Hunchback (HB), Giant (GT), Krüppel (KR) Knirps (KNI) and Sloppy-paired 1 (SLP1).

*hb*, *gt* and *Kr* are expressed in *T. minor* in patterns that mimic those of their orthologs in *D. melanogaster* embryos (Figure 4A–C,E–G). This is in contrast to AP patterning factors in the mosquito, in which there have been shifts in expression domains, and presumably changes in regulation of the downstream genes [16].

We were unable to clone the *kni*, *slp1* and *cad* genes from *T. minor*. In *D. melanogaster*, *bcd* RNAs are tethered to the anterior pole of the embryo, with BCD protein diffusing away from the pole to create a strong anterior to posterior gradient. BCD antibodies were not cross-reactive in *T. minor*, and we were unable to characterize the *T. minor* BCD gradient.

Key elements of the heart regulatory network are conserved between flies and vertebrates [17]. As we therefore expect this network to be conserved between the sepsid and *Drosophila* species, and our supply of *T. minor* embryos was limited, we did not examine the expression of heart regulators.

### Identification of Sepsid Enhancers

Since the sepsid and *Drosophila trans*-regulatory networks regulating *eve* expression appear to be similar, we reasoned that sepsid enhancers would contain similar collections of transcription factor binding sites as their *Drosophila* counterparts. In *D. melanogaster*, clusters of HB, CAD, KNI, KR, and BCD binding sites in the *eve* locus have been shown to correspond to known stripe enhancers [18]. We therefore examined the density of predicted HB, CAD, KNI, KR, GT and BCD binding sites across each fosmid sequence (Figure S3) and identified 18 candidate sepsid stripe enhancers (Table S4) (We recently generated GT *in vitro* binding data which was not available when the initial *D. melanogaster* work was carried out).

Each of these predicted enhancers contained a small number of short (20–30 bp) sequences conserved between sepsids and drosophilids, which established presumptive orthology with specific regions of the *D. melanogaster* genome. In essence, the binding site plots showed us where sepsid enhancers could be found, and the small islands of sequence conservation suggested their likely function.

We also identified putative *eve* muscle-heart enhancers (MHE) (Table S4) in the sepsid species by looking for short blocks (20–30 bp) of high similarity (>90%) that overlap functionally verified transcription binding sites from the *D. melanogaster* MHE in pairwise alignments between the *D. melanogaster* MHE and each of the sepsid intergenic regions.

### Function of Sepsid eve Enhancers in Transgenic *D. melanogaster* Embryos

**Figure 5:**
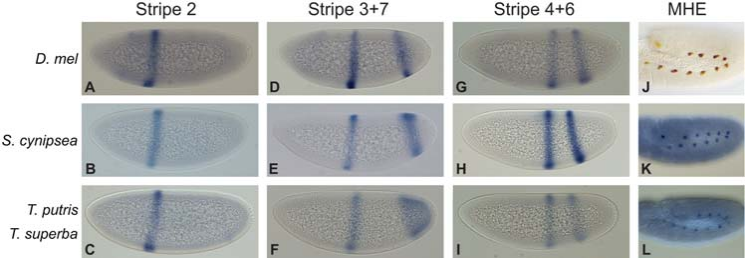
Sepsid *eve* enhancers drive conserved expression patterns in *Drosophila melanogaster* embryos. Expression patterns of *eve* stripe 2, stripe 3+7, stripe 4+6 and muscle-heart enhancers from sepsids *S. cynipsea*, *T. putris* and *T. superba* were compared to their *D. melanogaster* counterparts in transgenic *D. melanogaster* embryos by RNA *in situ* hybridization with digoxigenin-labeled antisense RNA probes against the reporter genes lacZ (A, D, G) and CFP (B,C,E,F,H,I,K,L), or staining with βGal antibodies (J). (A–C) Sepsid stripe 2 enhancers drive strong expression in an anterior stripe corresponding to *D. melanogaster* stripe 2. (D–F) Sepsid stripe 3+7 enhancers drive expression within the limits of *D. melanogaster* stripe 3 and 7, with additional expression in the posterior. (G–I) Sepsid stripe 4+6 enhancers drive expression within the limits of *D. melanogaster* stripes 4 and 6. (J–L) Sepsid MHE enhancers are expressed in metameric clusters in the dorsal mesoderm in stage, as in *D. melanogaster*. Embryos were imaged during cellularization and are oriented with anterior to the left and dorsal up.

We chose to test whether candidate enhancers from one species in each of the two sepsid clades were capable of driving expression in *D. melanogaster* embryos. Enhancer-reporter cassettes for each of these 8 constructs were introduced into the *D. melanogaster* genome via Phi-C31 phage-mediated targeted integration [19],[20]. Remarkably, despite their extensive sequence differences, all of the tested sepsid sequences drive very similar expression patterns to those driven by their orthologous *D. melanogaster* enhancers (Figure 5), although there are some small and intriguing differences. This confirms that these sepsid sequences are functional *eve* enhancers that, with their high degree of sequence divergence, represent markedly different examples of how to construct an *eve* enhancer.

The *D. melanogaster* minimal stripe 2 element drives expression in a single stripe in the stage 5 blastoderm from 63–57% egg-length through activation by broad anterior gradients of BCD and HB and localized repression by GT and SLP1 in the anterior and KR in the posterior [21] (Figure 5A; Table 2). The sepsid stripe 2 enhancers in the transgenics similarly drive expression from 62–55% egg-length (Figure 5B,C; Table 2). In 78% of embryos containing the *S. cynipsea* enhancer and 55% of embryos containing the *T. putris* enhancer, we observe expression in stripe 7 from the sepsid stripe 2 enhancers; similar behavior has also been observed for *D. melanogaster* stripe 2 constructs [21].

**Table 2:** Positions of borders and width of *eve* stripes driven by *Drosophila* and sepsid enhancers.

Enhancer	Border	*D. melanogaster*	*S. cynipsea*	*T. putris*
stripe 2	anterior	63±1.5	62±0.8	62±1.3
	posterior	57±1.2	55±0.9	54±1.1
stripe 3+7	anterior	53±1.1	54±1.4	53±1.1
	stripe 3 posterior	47±1.1	49±1.5	46±0.9
stripe 4+6	stripe 4 anterior	47±1.0	47±0.7	46±1.7
	stripe 4 posterior	40±1.0	40±0.8	40±2.2
stripe 4+6	stripe 6 anterior	30±1.5	32±1.1	30±1.9
	Stripe 6 posterior	22±1.9	25±0.9	24±2.0
stripe 2	stripe 7 anterior	−	21±1.4	21±1.5
	stripe 7 posterior	−	15±1.3	15±1.0
stripe 3+7	stripe 7 anterior	21±1.3	18±1.5	22±1.6
	stripe 7 posterior	12±1.3	9±1.0	0

Enhancer	Feature	*D. melanogaster*	*S. cynipsea*	*T. putris*
stripe 2	stripe 2 width	6±0.8	7±0.7	8±0.6
stripe 3+7	stripe 3 width	7±0.8	8±2.3	7±0.6
stripe 4+6	stripe 4 width	8±0.6	6±0.7	7±0.9
stripe 4+6	stripe 6 width	8±0.7	6±0.7	6±0.7
stripe 2	stripe 7 width	−	7±0.4	7±0.9
stripe 3+7	stripe 7 width	8±1.0	9±1.0	22±1.6

The *D. melanogaster* stripe 3+7 enhancer (Figure 5D) is broadly activated by dStat and Tailless (TLL) (stripe 7 only), and the two stripes of expression at 53–47% and 21–12% egg-length (Table 2) are carved out by domains of HB, KNI, and SLP1 repression [22]. Stripe 3 expression in the transgenics containing sepsid stripe 3+7 enhancers agrees well with *D. melanogaster* (Figure 5E,F; Table 2). The anterior border of stripe 7 corresponds to that in *D. melanogaster*, but in embryos containing either the *S. cynipsea* or *T. putris* stripe 3+7 element, stripe 7 expression extends posteriorly. Significantly, the stripe 3+7 enhancer has been inverted in the *Sepsis* species relative to the other sepsids and *Drosophila*. This strongly suggests that these enhancers are orientation-independent in their native genomic context.

The *D. melanogaster* stripe 4+6 enhancer drives expression in 2 stripes from 47–40% and 30–22% (Figure 5G). There is some evidence that stripe 4+6 expression is activated broadly by Dichaete and restricted to 2 stripes by HB and KNI repression, but the precise details of its regulation are less well understood [23],[24]. This pattern is reproduced in our transgenics, with expression from 46–40% and 31–25% egg-length (Figure 5H,I; Table 2).

In stage 11 *D. melanogaster* embryos, *eve* is expressed in laterally-symmetric, metameric pairs of pericardial cells in the dorsal mesoderm (Figure 5J) [25]. The *eve* MHE integrates activation and repression from multiple signaling pathways, including DPP and WG from the dorsal ectoderm and RAS in the dorsal mesoderm [26]. In addition, broad domains of TIN and TWI in the dorsal mesoderm activate expression. This metameric pattern is faithfully reproduced by the sepsid MHE enhancers (Figure 5K,L).

### Binding Site Composition and Organization in Sepsid Enhancers

That enhancers with minimal sequence conservation have conserved function suggests that they share some common features beyond primary sequence. In order to examine what these shared properties might be, we examined and compared the composition and organization of predicted transcription factor binding sites in all of the characterized *eve* enhancers. We restricted our analysis of each enhancer to those factors known to be involved in the activity of the particular enhancer.

**Figure 6:**
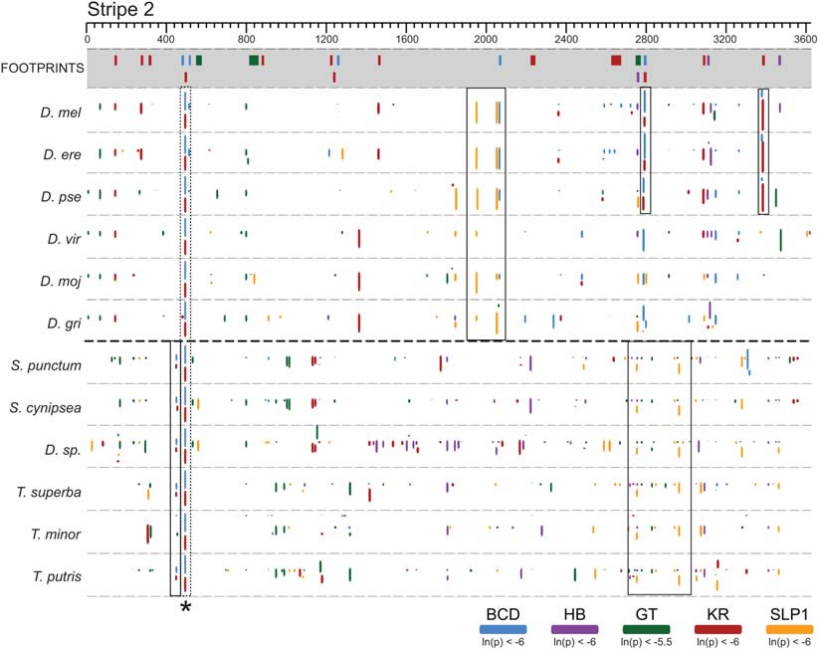
Extensive reorganization of binding sites between *Drosophila* and sepsid *eve* stripe 2 enhancers. Predicted binding sites for the five factors known to regulate expression from the *eve* stripe 2 enhancer in six *Drosophila* species [56] and six sepsid species. Sites were predicted independently in each species using PATSER [61] and mapped onto an MLAGAN [65] multiple alignment of the *eve* stripe 2 enhancer sequences. The height of the box representing each binding site is scaled by its PATSER p-value (taller boxes represent sites with higher predicted affinities). The top panel (grey shading) shows the positions of biochemically-verified (*in vitro* footprinting) binding sites [27]. Binding sites conserved within families are indicated by solid boxes. A BCD-KR site pair conserved across families is indicated by a dashed box. Alignment coordinates are indicated.

We aligned enhancer sequences from within each family, and plotted predicted transcription factor binding sites on these alignments (Figure 6). 92% of *D. melanogaster* binding sites are found in the same location in enhancers from other species within the closely related *melanogaster* subgroup (Table S3), 29% of sites are similarly conserved between *D. melanogaster* and the species of the *virilis*-*repleta* clade (Table S3). An average of 22% of sites are conserved between *S. cynipsea* and *Themira* species. The non-coding divergence between these two sepsid clades is similar to that between *D. melanogaster* and the *virilis*-*repleta* clade (Figure 3). This is likely an underestimate of the conserved sites within the sepsids as these are not minimal enhancers and thus should contain a larger portion of non-conserved background sites.

The lack of sequence similarity between families made nucleotide level alignment of sepsid enhancers to their *Drosophila* orthologs impossible. However, the previously described small blocks of high sequence conservation allowed us to orient and crudely align the sepsid and drosophilid enhancers to each other. In examining plots like this for all four enhancers, it was clear that few of the binding sites conserved within each family were conserved between families (Figure 6; Figure S4). Only 5% of *D. melanogaster* binding sites are conserved in pairwise comparisons with sepsid species, representing an additional 84% reduction in conserved sites compared to the *virilis*-*repleta* clade (Table S3).

However, we note that all of the highly conserved blocks contained at least one, and often several, highly conserved binding sites, and that most of these sites correspond to known *in vitro* footprints for the corresponding factor in *D. melanogaster* [27] (Figure S4).

### Conservation of Binding Site Composition

Most early embryonic enhancers in *D. melanogaster* contain unusually large numbers – compared to random non-coding sequence – of predicted binding sites for the factors involved in their regulation [18],[28], although the exact relationship between binding site density and function remains to be elucidated. Binding site density is conserved between enhancers in *D. melanogaster* and *D. pseudoobscura* [13],[14], but it is not clear how much of this conservation is due to selection to maintain binding sites, and how much is due to the overall high level of sequence conservation between *D. melanogaster* and *D. pseudoobscura*.

### Deep Conservation of Paired Binding Sites

Given the overall lack of sequence and binding site conservation between sepsid and *Drosophila* enhancers, we were particularly interested in the characteristics of the small sequence blocks that are conserved between the families. We noticed that all of these blocks contained overlapping or tightly spaced binding sites.

**Figure 7:**
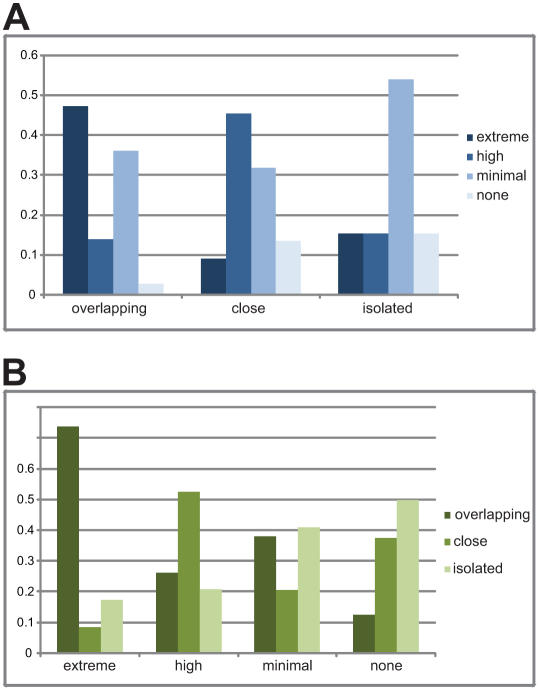
Evolutionary fate of binding sites is dependent on their proximity to other sites. Binding sites in the stripe 2, stripe 3+7, and MHE enhancers were classified as “overlapping” if they shared at least one base pair with a site for a different factor, “close” if the nearest base of another site (for a different factor) is within 10 bp, and “isolated” if neither condition is met. Binding sites in *D. melanogaster* were classified as non-conserved, minimally conserved (only within *melanogaster* subgroup), highly conserved (within 12 sequenced *Drosophila* species) and extremely conserved (12 *Drosophila* and 6 sepsids). (A) The distribution of conservation scores as a function of binding-site proximity shows overlapping and close sites are more likely to be highly or extremely conserved than isolated sites. (B) The fraction of each conservation category in different proximity groups again shows that extremely and highly conserved sites are strongly enriched for overlapping and close binding sites.

To analyze this more rigorously, we classified predicted *D. melanogaster* binding sites for footprinted factors in the *eve* MHE, stripe 2 and stripe 3+7 enhancers into four categories ranging from non-conserved (present only in *D. melanogaster* and its immediate sister taxa) to extremely highly conserved (present in *Drosophila* and sepsids). We then classified sites based on their proximity to other predicted binding sites: overlapping sites that share one or more bases with another binding site, neighboring sites that are within 10 bases of another site but do not overlap, and isolated sites. Overlapping sites are more often extremely conserved, close sites are more often highly conserved and isolated sites are more often minimally or non-conserved than expected by chance (Figure 7; p<0.007, p<0.01, p<.049, Chi-squared test). However, the number of sites is too small to detect relationships between conservation and the spacing of pairs of sites for specific factors.

## Discussion

We have demonstrated here that complex animal regulatory sequences can tolerate nearly complete rearrangement of their transcription factor binding sites without appreciably altering their transcriptional output. Thus, while the global organization of binding sites within regulatory sequences plays an important role in determining their function, strong evolutionary constraint to maintain expression patterns does not require the maintenance of any single binding site architecture. Despite this flexibility in the overall organization of regulatory sequences, our analysis of the small number of binding sites conserved between *Drosophila* and sepsid species suggests strong selection to maintain overlapping and adjacent pairs of binding sites.

Although *Drosophila* has been the subject of more extensive genome sequencing than any other animal genus, these observations were not evident from comparing the 12 sequenced *Drosophila* genomes. Only with the inclusion of species with similar development but substantially more highly diverged genomes did these properties emerge.

### Binding Site Turnover and the Regulatory Machinery

Our work extends in both the extent of divergence and number of enhancers examined the pioneering work on binding site turnover of Ludwig and Kreitman, who showed in a series of papers that the *eve* stripe 2 enhancer from other *Drosophila* species drives a stripe 2 pattern in transgenic *D. melanogaster* embryos despite the imperfect conservation of functional binding sites [5],[6],[8].

Although several examples of *Drosophila* regulatory sequence conservation over long evolutionary distances had been reported prior to Ludwig and Kreitman's work on *eve* stripe 2 [29],[30], *eve* regulation has become the preeminent model for the study of binding site turnover. It remains one of the few cases where observations of expression pattern conservation have been followed up with studies of functional complementation [7].

We have nearly doubled the evolutionary distance analyzed by Ludwig and Kreitman. Furthermore, in their comparisons the majority of binding sites were conserved, while our species sample has very few conserved binding sites. We have also generalized their observation to include additional enhancers responding to a different suite of transcription factors, including one (the MHE) active following gastrulation. Previous reports of the functional equivalence of divergent enhancers in *Drosophila* have involved blastoderm enhancers, leaving open the possibility that the observed binding site turnover was a byproduct of the syncitial nature of the early *Drosophila* embryo. Our data on the MHE demonstrates that extreme binding site turnover with functional conservation occurs in enhancers active in a cellular context.

A handful of isolated case studies support our findings. For example, the *tailless* enhancer from the house fly *Musca domestica* [31] and the *single-minded* enhancer from the mosquito *Anopheles gambiae* [32] drive similar patterns as their endogenous orthologs in *D. melanogaster* embryos despite having different organization of binding sites, and non-coding sequences from the human *RET* locus drive *ret*-specific expression in zebrafish despite the absence of detectable sequence similarity between human and zebrafish *RET* non-coding DNA [33]. Nonetheless, in each of these cases simple transcription factor “grammars” were conserved, offering a ready molecular explanation for the conserved function. No such grammar is as of yet apparent in the *eve* enhancers.

Such remarkable flexibility in the organization of enhancers suggests that the protein-protein and protein-DNA interactions that mediate the activity of developmental enhancers are not highly structured as, for example, is seen in enhanceosomes [34]. If they were, it is hard to imagine how such wildly different sequences could produce identical expression patterns in the same *trans*-regulatory context. The extent of binding site turnover is consistent instead with the recently proposed “billboard” model of enhancer activity in which enhancers contain multiple sub-elements that independently interact with cofactors and the basal machinery to dictate transcriptional output [35]–[37]. In proposing the billboard model, Kulkarni and Arnosti proposed that billboard enhancers would be more evolutionarily pliable than enhanceosomes, and suggested that the *eve* stripe 2 results from Ludwig and Kreitman were understandable if *eve* stripe 2 were a billboard enhancer [35]. Their model does not, however, predict how evolutionarily flexible billboard enhancers should be. Our discovery of extreme sequence and binding site divergence between functionally equivalent sepsid and *Drosophila* enhancers shows that they are extremely flexible, a fact that must be accounted for in future models of enhancer activity.

However even billboard enhancers are not infinitely flexible. One remarkable aspect of enhancer evolution is that despite the clearly frequent repositioning or replacement of transcription factor binding sites within enhancers, the enhancers themselves remain fairly compact. There must, therefore, be selection to keep the different sub-elements that contribute to an enhancer's output within the one to two kilobase span of a typical enhancer. This spatial constraint implies some functional interaction between enhancer sub-elements not currently captured by the billboard model.

### The Importance of Paired Sites in Gene Regulation

Given the extent of non-coding divergence between *Drosophila* and sepsids across most non-coding DNA, we were surprised to observe small islands of very strong sequence conservation. Our finding that there is a significant enrichment of overlapping or adjacent binding sites within conserved blocks lends evolutionary support to long-standing suggestions of the importance of direct competitive and cooperative interactions between bound transcription factors.

Numerous studies have demonstrated that appropriate regulation of the eve stripe enhancers (and other enhancers) relies on the close proximity of multiple binding sites for both activators and repressors [21], [36], [38]–[41].

Of the 12 footprinted BCD, HB, KR, and GT sites in the minimal stripe 2 element, 8 fall into 2 clusters of about 50 base pairs each containing overlapping activator (HB or BCD) and repressor (KR or GT) sites. In transient transfection experiments using these binding site clusters, BCD and HB dependent activation was repressed by DNA binding of GT or KR, consistent with the short-range repression mechanisms of quenching or competition [40]. Knirps also mediates short-range repression in a range of 50–100 bp through quenching or direct repression of the transcriptional machinery when bound near a promoter [42].

Similarly, HB and BCD co-expression in transient transfection experiments results in multiplicative activation of a reporter construct containing a subset of the *eve* minimal stripe 2 element [40]. Mutation of single activator sites in the minimal stripe 2 element results in a significant reduction in expression, again suggesting that HB and BCD bind cooperatively to this enhancer [21].

The local quenching and cooperativity models predict that binding sites in close proximity to each other should be under strong purifying selection to remain close to each other. Under the generally accepted model of binding site turnover, sites are lost in one region of an enhancer when new mutations create a complementary site elsewhere in the same enhancer. The appearance of new sites is the rate-limiting step as there are more mutational steps required to create a new site from random sequence than to destroy an existing site. Since random mutations are far less likely to produce pairs of adjacent sites than single sites, we expect functionally linked pairs of sites to be subject to far lower rates of binding site turnover. In contrast, if binding site turnover is driven by base substitutions, we expect functionally independent sites that are adjacent or even partially overlapping to have essentially the same rates of binding site turnover as isolated sites. The conserved blocks we observed between sepsids and *Drosophila* were generally larger than individual sites, as has been previously reported within *Drosophila* [43], consistent with the former model. Our observation that proximal sites are preferentially conserved additionally supports their direct functional linkage.

However, we note that insertions and deletions are a major source of sequence variation in *Drosophila*, with *D. melanogaster* having a strong deletion bias [44] and deletion is thought to contribute significantly to binding site turnover [45]. Taking this into account, we expect to observe reduced turnover in even functionally independent binding sites if they are overlapping or adjacent, as some fraction of the deletions that would remove a binding site with a complementary site elsewhere would also affect adjacent, and presumably uncompensated sites. These deletions would be subject to purifying selection, and the rate of turnover for the proximal sites would be reduced. Assessing whether such an effect could explain our observation requires more data on relative rates of nucleotide substitution and insertion and deletions of different sizes in sepsids, which will be accomplished with the sequencing of sepsid genomes.

We can, however, test the significance of our observation directly. The linked function model predicts that the paired binding sites we observe to be conserved between families should be more sensitive to manipulations that alter the spacing between the sites than paired binding sites that are not conserved.

### Variation in *eve* Stripe Patterns

Though expression of the sepsid *eve* enhancers in *D. melanogaster* embryos is qualitatively very similar to the patterns driven by the *D. melanogaster* enhancers, there are subtle and interesting differences. Expression of stripe 7 exhibits the most variability across all enhancers in transgenics, including those enhancers from *D. melanogaster*. It was previously observed that stripe 7 is weakly expressed in *D. melanogaster* stripe 2 transgenics, and stripe 7 expression is weaker than the endogenous stripe in stripe 3+7 transgenics [21],[22],[40]. We frequently observed stripe 7 expression in all our non-*Drosophila* stripe 2 transgenics, and stripe 7 expression did not perfectly recapitulate endogenous expression, suggesting that regulatory information specifying this stripe is distributed across the upstream region, thus challenging the model of enhancer modularity in agreement with [46]. Information may be more diffusely spread across the locus in sepsids, resulting in missing information in our discrete cloned enhancers, in which case the native *D. melanogaster* pattern should be more accurately reproduced by cloning a larger regulatory region. Alternately, there could be changes within the non-*Drosophila* enhancers which result in expression differences in *D. melanogaster* despite conserved native *eve* expression, suggesting co-adaptation of each enhancer and its native *trans* environment.

### Species Choice and the Value of More Distant Comparisons

We began this study seeking taxa that were significantly more diverged from *D. melanogaster* than any *Drosophila* species, but which had sufficiently conserved *cis*-regulatory networks that their enhancers would have similar function to their *D. melanogaster* counterparts. Our choice of sepsids was guided by their relatively close – but not too close – position to *Drosophila* on published trees of Diptera [47], by their relatively similar morphology suggestive of similar developmental mechanisms, and by practical considerations such as genome size and availability.

We have now shown that the extensive sequence divergence between sepsids and *Drosophila* was not accompanied by extensive differentiation of early embryonic patterning mechanisms. Thus sepsids provide a valuable model for comparative analysis of *Drosophila* embryology and developmental *cis*-regulation. We were also able to establish a colony of sepsids (*T. minor*) in the lab from flies caught locally, and collect embryos for the developmental gene expression and morphology data presented here. Based on our experience, we believe that more extensive embryological and molecular work with sepsids is very feasible, although some may find the need to provide the colonies with fresh cow dung objectionable.

The additional sequence divergence has enabled us to reach two important conclusions that could not be obtained in analyses of the 12 sequenced *Drosophila* genomes. Previous analyses of binding site turnover in *Drosophila* revealed substantial numbers of conserved binding sites within the genus, leaving open the question of whether these sites represented an imperturbable core necessary for enhancer function, or if there had simply not been sufficient divergence time for mutation to generate alternative configurations. We have now largely answered this question, at least for the *eve* enhancers – there does not appear to be an imperturbable core of sites at the level of overall enhancer organization.

Although binding site conservation in *Drosophila* has been extensively studied, our observations about the relationship between conservation and binding site proximity were never described because this pattern was simply not evident in examinations of the multitude of conserved binding sites across the *Drosophila* genome. This relationship only became apparent when we observed just how striking the conservation of a small subset of sites was.

More generally, this study highlights the value of the infrequently studied (at least by molecular biologists) Dipteran species outside of the genus *Drosophila*. It also points to a general strategy for dissecting the still elusive molecular mechanisms of enhancer function in which genome sequencing and functional studies are combined to catalog the diverse ways in which regulatory sequences with common function can be generated. Our initial foray into this domain has yielded exciting and unanticipated results. With the cost of genome sequencing plummeting, and with great improvements in *Drosophila* transgenesis, we expect this approach to be even more productive in the years to come.

## Methods

### Specimens

*Sepsis punctum*, *Sepsis cynipsea*, *Themira superba*, *Themira putris and Dicranosepsis sp.* stocks were maintained in the Evolutionary Biology Laboratory at the National University of Singapore. *Themira minor* cultures were established at LBNL from specimens collected at McKinley Park in Sacramento, CA. Samples for genome sizing and genomic DNA isolation were flash-frozen adult flies.

### Genome Size Determination

Genome sizing methods were adapted from [48]. Five adult heads for each species were dissected into 1.5 mL of Galbraith buffer on ice, homogenized with 15 strokes of an A pestle in a 15 mL Kontes Dounce tissue homogenizer, and filtered through 30 um nylon mesh. *T. superba* heads were combined with 5 *D. virilis* heads before homogenization. 7 uL of 1∶10 chicken red blood cells (diluted in PBS) and 50 uL of 1 mg/mL propidium iodide were added and samples were stained for 4 hours rocking at 4 degrees in the dark. Mean fluorescence of co-stained nuclei was quantified on a Beckman-Coulter EPICS XL-MCL flow cytometer with an argon laser (emission at 488 nm/15 mW power). The propidium iodide fluorescence and genome size of *Gallus domesticus* (red blood cell standard, 1,225 Mb) were used to calculate the unknown genome sizes. For *T. superba, D. virilis* at 328 Mb, was used as a second internal standard.

### Fosmid Library Preparation

High molecular weight genomic DNA was obtained from approximately 500 mg of frozen adult flies using the Qiagen 500/G Genomic-tip protocol for isolation of genomic DNA from flies (Qiagen Cat. No. 10262). Fosmid libraries were generated according to the Fosmid (40 kb) Library Creation Protocol developed at the DOE Joint Genome Institute (http://www.jgi.doe.gov/sequencing/protocols/prots_production.html) with the following modifications. DNA was end-repaired without hydro shearing, phenol-extracted, and precipitated a second time after gel-purification to increase cloning efficiency. Ligation reactions were incubated overnight at 16°C with T4 DNA ligase then packaged according to the JGI protocol. All libraries are at approximately 5× coverage with an average insert size of 39.5 kb.

### Library Screening

Species specific sequence for target genes was obtained by degenerate PCR with primers designed based on *Drosophila* protein sequences, with additional fly sequences used where available. 40 bp overlapping oligonucleotide probes were synthesized by Klenow extension of 24 bp oligos overlapping by 8 bp with radiolabeled dATP/dCTP. Oligos were designed against target gene regions with 50–55% GC and no matches to known PFAM domains. Overgo probes were hybridized in pools of 6–10 probes to high density colony array filters at 60 degrees C overnight as described in [49] and visualized on a Molecular Dynamics Storm 860 phosphorimager. Positive clones were isolated and fosmid DNA was extracted and printed in 12×8 arrays on nylon membranes for hybridization with single overgo probes, protocol as above. 1–3 fosmid clones for each gene in each species were selected by EcoRI and BglII restriction mapping from final dot blot positives and were shotgun sequenced.

### Sequencing and Assembly

Selected fosmids were subcloned and sequenced at the Joint Genome Center; protocols are available at http://www.jgi.doe.gov/sequencing/protocols/prots_production.html.

Chromatograms were reanalyzed using PHRED v0.020425.c [48],[50],[51] using the phredPhrap Perl script supplied with the CONSED distribution to call bases and assign quality scores. The ARACHNE assembler [52],[53] was then used to build scaffolds (Table S2). After assembly, contigs from fosmids tiling across a given locus for a particular species were further merged by alignment using BLAT [54] (version 25; run with default parameters). Where matches exceeded 98% identity and extended to within 100 basepairs of either: a) both ends of a single contig, or b) one end of both contigs, one of the two sequences for the match region was chosen at random to construct a single representative sequence for the entire region, despite heterozygosity in fosmid libraries.

Fosmid sequences and combined locus sequences are available as Dataset S1.

### Annotation

Protein-coding gene annotation of the fosmids was performed with reference to the Flybase *D. melanogaster* 4.3 annotations. *D. melanogaster* translations were compared to the fosmid sequences translated in six frames using BLASTX. GeneWise [55] was used to construct gene models on scaffolds having hits with e-value ≤1e−10, with the query translation as template. Gene models were then filtered by requiring that the model translation find the original *D. melanogaster* query translation among the top hits in a reciprocal BLASTP search against the *D. melanogaster* translation set (e-value threshold 1e–10).

### Coding and Non-Coding Trees

We obtained established phylogenies of *Drosophila* [56] and Sepsidae [57]. Branch lengths for coding regions were determined using nucleotide sequence aligned in amino acid space with T-Coffee [58]. Codeml from the PAML package (version 3.13d, codon frequencies estimated from base frequencies [F3×4], no clock, single dN/dS across all branches estimated with a starting value of 0.4, transition/transversion ratio estimated starting at 2) was used to estimate branch lengths over 10 sequenced *Drosophila* species (not including *D. simulans* or *D. sechellia*) and the 6 sepsid species reported here for orthologs of seven genes (*bcd*, *CG8386*, *CG9119*, *eve*, *odd*, *stumps*, *zen*) independently, as well as for the concatenation of all seven, and for the seven arrangements of all but one gene. The 15 resulting trees were compared both by visual inspection, and RMSD of branch lengths. Single gene trees constructed from alignments with 115 or fewer informative positions (*eve*, *CG9119*) estimated many zero-length internal branches and higher RMSD from the full concatenation (up to 390% average branch length), however no leave-one-out tree deviated from the seven gene concatenation by more than 15% of average branch length, suggesting that no single gene dramatically distorts the overall estimate of branch lengths in the full set concatenation. We therefore report final results for per-codon rate estimates of the full concatenation of 1566 informative positions.

Phylogenies of noncoding regions surrounding the *eve* gene were estimated in each family separately using baseml from then the PAML package [59] (Model: HKY, transition/transversion ratio estimated as above, alpha estimated starting at 0.5). A total of 966 sites in *Drosophila* and 958 sites in Sepsid alignments proved informative in upstream and downstream regions combined. Final estimates from these upstream + downstream concatenations in each family are reported as per-base rates.

### Determination of Endogenous Expression Patterns in Sepsids

Fresh cow dung was obtained from free-ranging, grass fed, and antibiotic-free Milking Shorthorn cows (*Bos taurus*) in the Tilden Regional Park in Berkeley, CA. Resting cows were approached with caution and startled by loud shouting, whereupon the cows rapidly stood up, defecated, and moved away from the source of the annoyance. Dung was collected in ZipLoc bags (1 gallon), snap-frozen and stored at −80 C. Dung aliquots were thawed at 4 C and moistened slightly before use.

*T. minor* embryos were collected at room temperature in a 100 mm petri dish of fresh cow dung. Embryos were removed from the top layer of dung under a dissecting microscope then filtered through course mesh to remove grass and debris. Fixation was as previously described for *D. melanogaster* in 50% fixation buffer (1.3× PBS, 66 mM EGTA pH 8.0) containing 9.25% formaldehyde [21]. 500–1000 bp of coding sequence for each gene were amplified from genomic DNA by degenerate PCR and cloned into the pGEM-T-Easy vector, amplified with M13 forward and reverse primers, and gel-purified with Qia-quick PCR columns. 4 uL of product were used in 20 uL transcription reactions with digoxigenin-11-UTP as described by the manufacturer (Roche DIG RNA Labeling Kit, Cat. No. 11 175 025 910). Probes were then incubated in 100 uL of 1× carbonate buffer (120 mM Na2CO3, pH 10.2) for 20 minutes, and reactions were stopped by addition of 100 uL stop solution (0.2 M NaOAc, pH 6.0). Probes were precipitated with 8 uL of 4 M LiCl and 600 uL EtOH then resuspended in 1 mL hybridization buffer. Hybridizations were performed as described previously with 18–20 hour hybridizations [60]. Embryos were imaged on a Nikon Eclipse 80*i* scope equipped with a Nikon Digital Sight DS-U1 camera.

### Enhancer Prediction

We picked regions of the fosmid to test for enhancer activity based on manual inspection of two types of data: (1) *D. melanogaster* enhancers mapped to each fosmid sequence via pairwise alignments, and (2) conservation of putative binding sites for BCD, CAD, KR, KNI, GT, HB. We computed pairwise LAGAN (Brudno et al. 2003) alignments of each sepsid fosmid to all of the other sepsid fosmids and the *eve* locus (defined as 10 kb upstream and downstream of the annotated *eve* protein-coding gene). In all cases, short blocks of high sequence similarity between *D. melanogaster* enhancers and the sepsid fosmids allowed us to determine the rough location of the likely sepsid enhancer. We used PATSER [61] and position weight matrixes for BCD, CAD, KR, KNI and HB from [18] and GT from data in [27] to predict sites for each factor across each fosmid using a ln(p-value) cutoff of −6. We assigned a conservation score to each site equal to the number of species in which a site for the same factor was predicted at an overlapping position in the pairwise alignment of the species. We examined the mean conservation score in 100 bp windows surrounding each mapped enhancer and selected boundaries for tested fragments to include regions of high single genome and conserved site density surrounding the region mapped from *D. melanogaster*. In the *Themira* clade, we tested stripe enhancer predictions from *T. putris* and the MHE prediction from *T. superba* as initially there was insufficient flanking sequence to recover all enhancers from the same species.

### Generation of *D. melanogaster* Transgenics

Enhancers were cloned into either the NotI or BglII site in pBΦY-ayeCFP vector (modified from pBDP-Gt81, kindly provided by Barret Pfeiffer). Reporter constructs were injected into the *D. mel* attP2 landing pad strain [20] by Genetic Services, Inc. Injection survivors were pooled and red-eyed progeny were screened from the F1 generation. Integration was confirmed by two PCR reactions, one which amplifies across the cassette integration site (fw: CGGCGGCAACCCTCAGCGGATG; rv: GCGAAGAGATAGCGGACGCAGCGG) and one which amplifies the enhancer within pBΦY (fw: AAATAGGGGTTCCGCGCACAT; rv: CCCCGCGCCCTTTTATACCG). *S. cynipsea* stripe 2 was confirmed with the cassette integration primers and primers that amplify within the enhancer (fw: TGGCACAAGAGCGCCTCGAA; rv: GCGAGCCCCTTTTCCGTTGG).

### Imaging of Transgene Expression Patterns

*D. melanogaster eve* stripe 2 and stripe 3+7 transgenic lines were kindly provided by Steven Small [21],[22] and *D. melanogaster* stripe 4+6 from Miki Fujioka [23]. For the stripe enhancers, transgenic embryos were collected for 2 hours then aged for 2 hours at room temperature. For the MHE, embryos were collected for 6 hours and aged 4 hours. Fixation, CFP and lacZ probe synthesis, hybridization conditions and microscopy were as described above.

### Sequence and Binding Site Conservation Metrics

Pairwise BLASTZ Score: BLASTZ [15] was run with default parameters except for MSP cutoff (K) of 1800, outputting but not extending chains (C = 1). BLASTZ hits can occur on either strand, and to any position in the other sequence, thus opposite strand matches (outside of the described inversion) and “non-local” matches (i.e. those to sequences far from the orthologous region defined by the synteny of matches overall) are a straightforward internal description of the precision of this method, and the cutoff selected. The chosen cutoff was selected in order to preserve hits that overlapped the “conserved cores” found by MLAGAN in each enhancer, while minimizing the number of minus strand and non-syntenic hits. BLASTZ alignments were generated for *D. melanogaster* enhancers in the eve locus against the nine *Drosophila* species and six Sepsid species shown in Table S4 and Table S5. The pairwise BLASTZ score is defined as the product of percent identity and the total length of HSP chains as reported by BLASTZ.

*D. melanogaster* binding sites in BLASTZ hits: Using BLASTZ alignments calculated above, we tabulated the number of binding sites predicted by PATSER in *D. melanogaster* enhancer sequences that occurred within BLASTZ aligned regions. Numbers reported reflect the total number of binding sites for factors in each enhancer as follows: HB, KNI, DSTAT in the stripe 3+7 enhancer; BCD, HB, GT, KR, SLP1 in the stripe 2 enhancer; TWI, PNT, PAN, MED, MAD, TIN in the muscle-heart enhancer; HB, KNI in the stripe 4+6 enhancer. A p-value cutoff of ln(p-value) <−6 was imposed on PATSER output.

Conserved binding sites in BLASTZ hits: We further tabulated the number of binding sites in a given *D. melanogaster* enhancer that fall within a BLASTZ HSP, in which the aligned sequence from the comparison species also contained an above-cutoff site prediction for the same transcription factor. In order for a site to be called “conserved” in a species pair, the comparison species binding site must overlap the *D. melanogaster* site by at least 1 bp.

Conserved binding sites in multiple alignment: MLAGAN was used to compute multiple alignments of the four enhancers listed above (stripe 2, stripe 3+7, stripe 4+6 and MHE) with default alignment parameters. Pairwise comparisons between *D. melanogaster* and each other species in an alignment were conducted as follows: each binding site prediction in *D. melanogaster* calculated as above was categorized as conserved in that species if a binding site better than ln(p-value) <−6 was present aligned within 5 bp of the boundaries of the site prediction in *D. melanogaster* (see alignment error correction, binding site dynamics in paper methods).

### Binding Site Dynamics Analysis

Binding sites were predicted in each species for each experimentally determined enhancer with published DNaseI footprints (stripe 2, stripe 3+7 and the Muscle Heart Enhancer) using PATSER [61] (version 3e) with a range of ln(p-value) cutoffs from −5 to −7. Position Weight Matrices for factors known to regulate expression driven from each enhancer were drawn from [61], except for GT (N. Ogawa and M.D. Biggin, unpublished) DSTAT [62], PAN/dTCF, PNT, and TIN [63] and TWI (D. Pollard, unpublished). PATSER was run with a GC content for each enhancer calculated from the entire *eve* locus in that species. *D. melanogaster* site predictions scoring above the cutoff were categorized into one of the following categories: “overlapping” if they overlap an above-cutoff site for a different factor by at least one basepair, “close” if the nearest base of another site (for a different factor) is within 10 bp, and “isolated” if neither condition is met Analyses described here use ln(p-value) <−6; results were robust to p-value cutoffs over the range described above (data not shown). Next, binding site predictions for each species were mapped onto a multiple alignment of all 18 species (12 *Drosophila* and 6 Sepsids) generated using MLAGAN (Brudno et al. 2003) (version 2.0, default run parameters). Finally, for each *D. melanogaster* site, the nearest aligned bases in each other species (plus/minus 5 bp for alignment error) were evaluated for presence of a binding site for the same factor. Three clades of increasing evolutionary distance were considered: “minimal” (*melanogaster* subgroup), “high” (all sequenced *Drosophila*), or “extreme” (12 *Drosophila* and 6 Sepsids). A given site in *D. melanogaster* was categorized by the largest clade in which it is conserved, where conservation is defined as presence in at least 80% of the species in that group (4/5 in subgroup, 10/12 in *Drosophila*, 15/18 in all species considered here). Thus, each binding site in *D. melanogaster* is categorized both by proximity to other binding sites in *D. melanogaster*, and by evolutionary stability across increasingly divergent species groups. Tabulations of these two properties were examined for relatedness by G-test of independence with Williams' correction for small sample size [64].

### Binding Site Plots

Enhancers were chosen for this analysis and those described above based on previously available data, specifically regarding the location of binding sites for important regulators. We analyzed eve enhancers for which DNase I footprinted *in-vitro* binding sites had been determined, and each such enhancer examined the minimal sequence interval that mediated the complete expression pattern, extended to include all footprinted binding sites for relevant regulators. Binding site predictions for each enhancer, as calculated above, were plotted in alignment position coordinates for each enhancer described here. Alignment position coordinates for binding site matches that overlapped a gap (in the species in which the site was predicted) were plotted at the midpoint of the gap. P-value cutoffs above which to plot glyphs for each factor were chosen independently from among predictions over the range described above (ln(p-value) between −5 and −7 in steps of 0.5) in order to maximize concordance with known DNase I footprinted binding sites in the *D. melanogaster* sequence for each enhancer. The height of each glyph is proportional to the score of that site prediction, and heights for the top scoring site match for each factor are normalized across all factors plotted in that enhancer.

## Supporting Information

Figure S1 Protein coding genes flanking the sepsid *eve* loci. The *eve* locus for each sepsid species is shown along with predicted orthologs of protein-coding genes from *D. melanogaster*. The displayed loci consist of a single scaffold, except for *S. punctum* and *D. sp.* for which multiple scaffolds were aligned and merged. Gene models were constructed using GeneWise [55] on scaffolds having BLASTX hits to *D. melanogaster* translations with e-value ≤1e−10.
(0.41 MB EPS)

Figure S2 Developmental timecourse of *Themira minor*. DIC images of fixed *Themira minor* embryos illustrate the major morphological stages of embryological development. Stages were determined according to Campos-Ortega & Hartenstein, 1985. The length of embryogenesis is similar in *Themira minor* and *D. melanogaster* at 25°C, with developmental stages corresponding closely and no obvious cases of heterochrony.
(1.51 MB EPS)

Figure S3 Enhancer prediction at the *eve* locus. Mean percent identity and mean percent gapped bases were calculated in 100 bp non-overlapping windows from all pairwise LAGAN alignments. Binding sites for BCD, CAD, KR, KNI, GT and HB were predicted in each fosmid using PATSER (ln (p)<−6). A conservation score was assigned to each site equal to the number of species in which a site for the same factor was predicted at an overlapping position in the pairwise alignment of the species. Single genome counts, conserved counts, and gapped counts of binding sites were calculated in 50 bp non-overlapping windows. Single genome counts and conserved counts of predicted binding sites in regions mapping to known *D. melanogaster* enhancers were predominately used for defining borders of enhancer predictions. Blue boxes show the mapped locations of *D. melanogaster* enhancers. Shaded grey boxes show the locations of tested enhancers.
(3.92 MB EPS)

Figure S4 Fine-scale sequence and binding site heterogeneity for stripe 3+7, stripe 4+6 and the MHE. Predicted binding sites for factors known to regulate expression from the (A) *eve* stripe 3+7, (B) stripe 4+6 and (C) muscle heart enhancers in six *Drosophila* species [56] and six sepsid species. Sites were predicted independently in each species using PATSER [61] and mapped onto an MLAGAN [65] multiple alignment of the eve enhancer sequences. The height of the box representing each binding site is scaled by its PATSER p-value (taller boxes represent sites with higher predicted affinities). The top panel (grey shading) shows the positions of biochemically-verified (in vitro footprinting) binding sites [27]. The indicated coordinates are for the multiple-alignment, which is longer than individual enhancers due to the high frequency of alignment gaps.
(2.24 MB PDF)

Table S1 Sequenced fosmids.
(0.04 MB DOC)

Table S2 *even-skipped* containing scaffolds or fused scaffolds used in analyses.
(0.03 MB DOC)

Table S3 Pairwise sequence and binding site comparisons.
(0.10 MB DOC)

Table S4 Predicted and tested sepsid enhancers.
(0.09 MB DOC)

Table S5 Analyzed *Drosophila* enhancers.
(0.12 MB DOC)

Dataset S1 Fosmid and locus sequences.
(0.18 MB GZ)
