# Supplementary material for: Sepsid even-skipped Enhancers Are Functionally Conserved in Drosophila Despite Lack of Sequence Conservation
Source: PLoS Genet. 2008 Jun 27;4(6):e1000106. doi: 10.1371/journal.pgen.1000106 (PMC2430619; doi:10.1371/journal.pgen.1000106)
Supplement: Figure S4 — Fine-scale sequence and binding site heterogeneity for stripe 3+7, stripe 4+6 and the MHE. Predicted binding sites for factors known to regulate expression from the (A) eve stripe 3+7, (B) stripe 4+6 and (C) muscle heart enhancers in six Drosophila species [56] and six sepsid species. Sites were predicted independently in each species using PATSER [61] and mapped onto an MLAGAN [65] multiple alignment of the eve enhancer sequences. The height of the box representing each binding site is scaled by its PATSER p-value (taller boxes represent sites with higher predicted affinities). The top panel (grey shading) shows the positions of biochemically-verified (in vitro footprinting) binding sites [27]. The indicated coordinates are for the multiple-alignment, which is longer than individual enhancers due to the high frequency of alignment gaps. (2.24 MB PDF) [file pgen.1000106.s004.pdf]

A

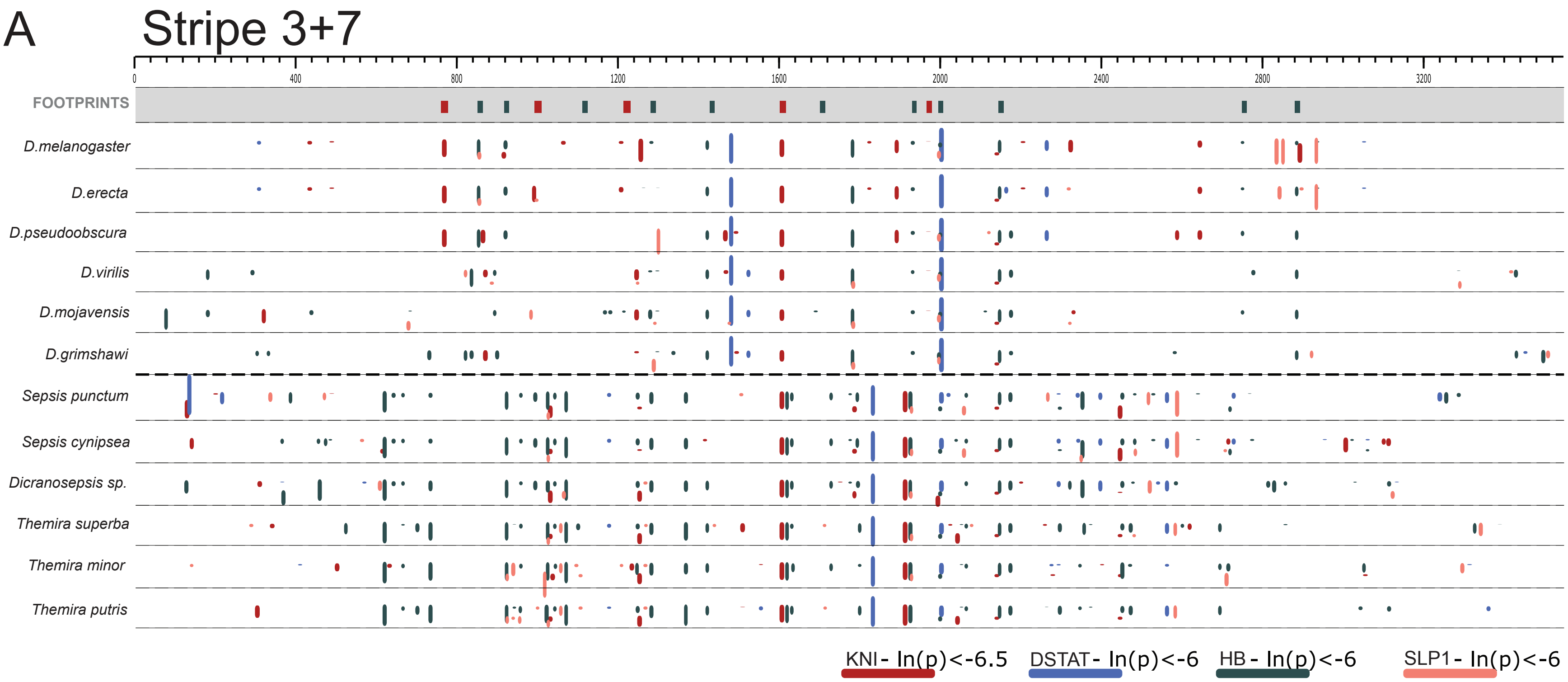

Supplementary Figure 4

B

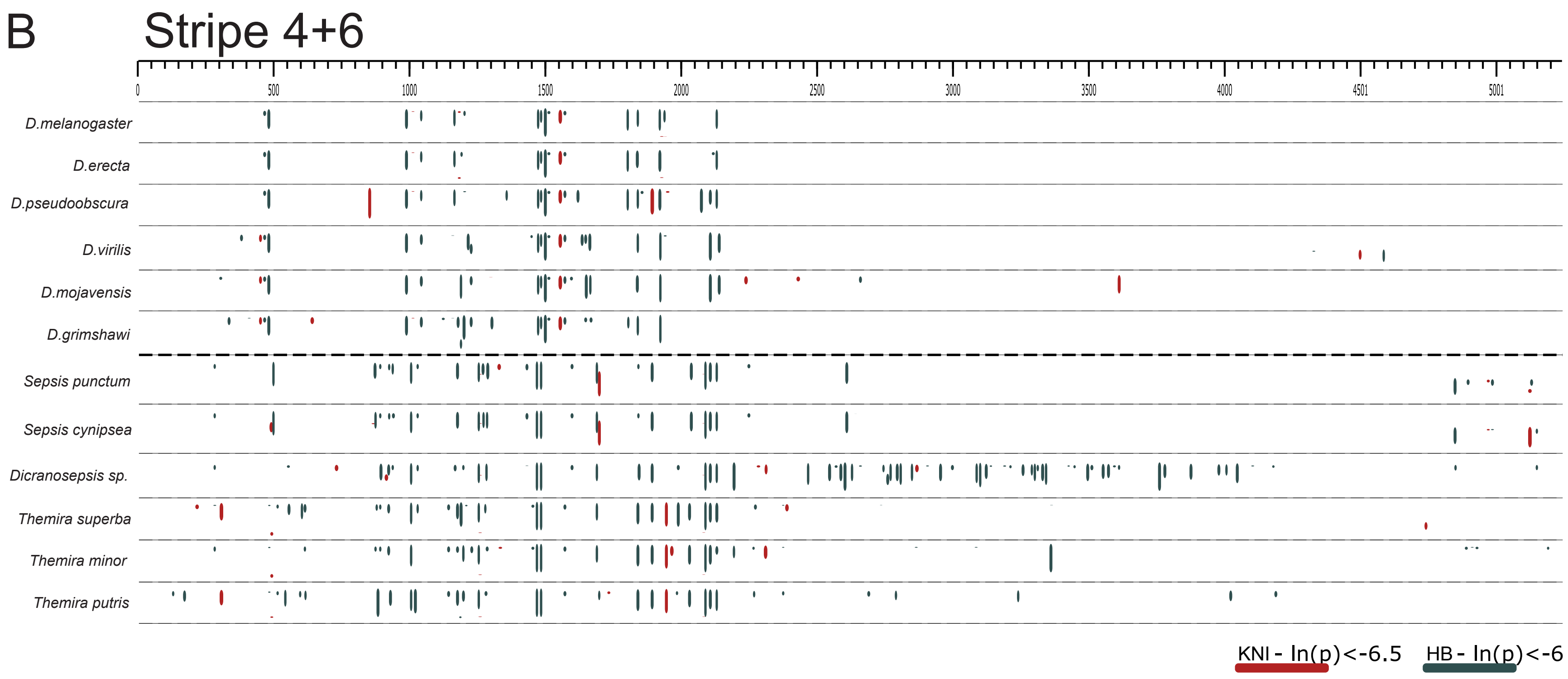

Supplementary Figure 4

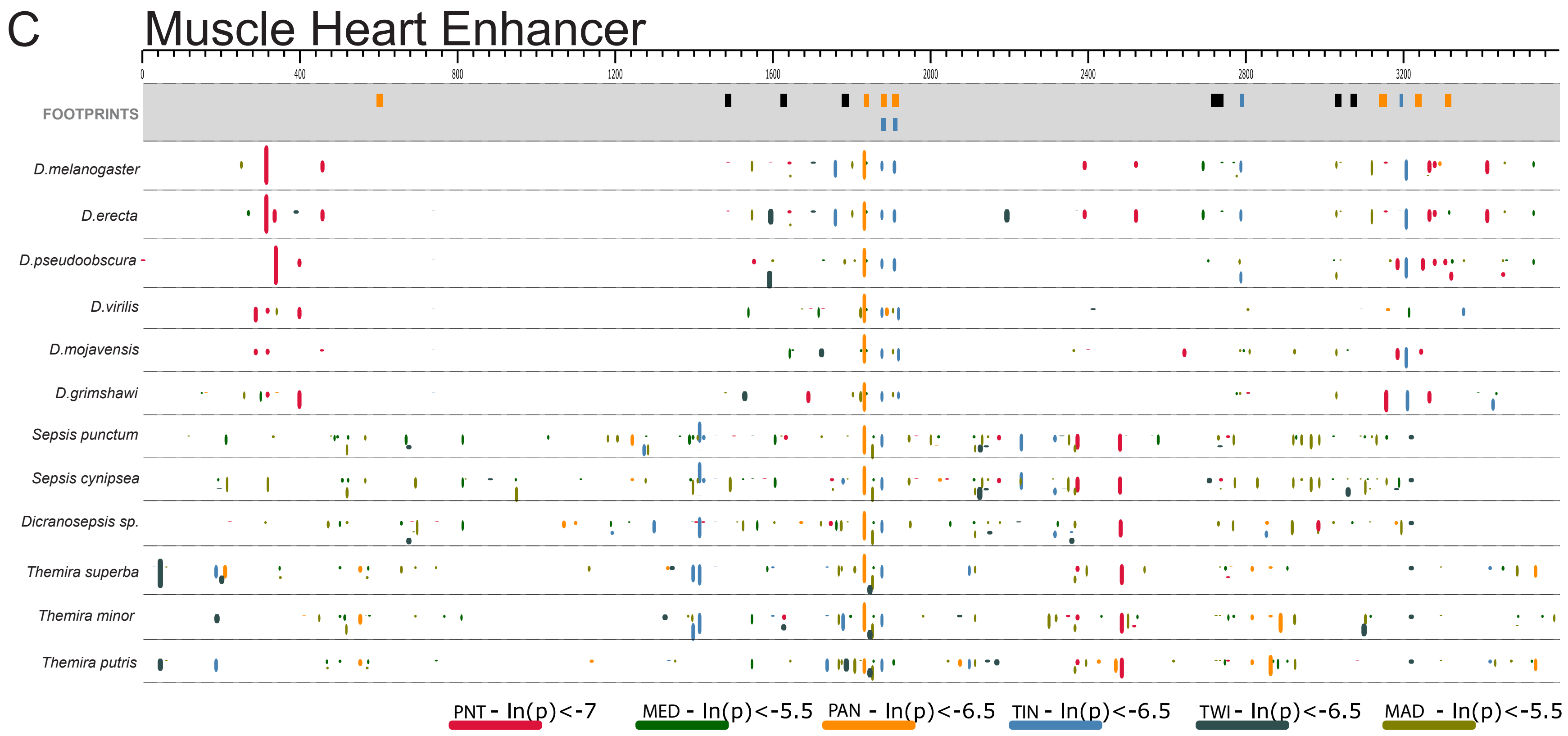

Supplementary Figure 4
