# Supplementary material for: Sepsid even-skipped Enhancers Are Functionally Conserved in Drosophila Despite Lack of Sequence Conservation
Source: PLoS Genet. 2008 Jun 27;4(6):e1000106. doi: 10.1371/journal.pgen.1000106 (PMC2430619; doi:10.1371/journal.pgen.1000106)
Supplement: Table S1 — Sequenced fosmids. (0.04 MB DOC) [file pgen.1000106.s005.doc]

Table S1. **Sequenced fosmids**

| **Species** | **Fosmids** | **Assembly Size** | **Number of Scaffolds** | **Reads** | **Coverage** | **GenBank**  **Accession** |
| --- | --- | --- | --- | --- | --- | --- |
| *T. putris* | BPGF | 39,600 | 3 | 768 | 14.6 | EU675300 |
| *T. minor* | FSIG | 52,494 | 11 | 768 | 10.4 | EU675306 |
|  | FSIH | 33,115 | 1 | 768 | 14.6 | EU675307 |
| *T. superba* | BPYI | 34,960 | 1 | 761 | 15.0 | EU675303 |
| *D. sp.* | BPWG | 35,915 | 4 | 768 | 15.1 | EU675301 |
| *S. cynipsea* | BUXF | 45,850 | 3 | 766 | 13.1 | EU675304 |
| *S. punctum* | BPXI | 39,012 | 4 | 768 | 7.7 | EU765302 |
|  | FCFB | 37,366 | 2 | 758 | 15.5 | EU686389 |
