# Supplementary material for: Sepsid even-skipped Enhancers Are Functionally Conserved in Drosophila Despite Lack of Sequence Conservation
Source: PLoS Genet. 2008 Jun 27;4(6):e1000106. doi: 10.1371/journal.pgen.1000106 (PMC2430619; doi:10.1371/journal.pgen.1000106)
Supplement: Table S2 — even-skipped containing scaffolds or fused scaffolds used in analyses. (0.03 MB DOC) [file pgen.1000106.s006.doc]

Table S2. ***Even-skipped* containing scaffolds or fused scaffolds used in analyses**

| **Species** | **Source** | **Size** |
| --- | --- | --- |
| *Themira putris* | EU675300 scaffold 0 | 36,229 |
| *Themira minor* | EU675306 scaffolds 0, 1, 2 | 47,965 |
| *Themira superba* | EU675303 scaffold 0 | 34,960 |
| *Dicranosepsis sp.* | EU675301scaffold 0 | 32,812 |
| *Sepsis cynipsea* | EU675304 scaffold 0 | 43,036 |
| *Sepsis punctum* | EU675305 scaffold 0 EU686389 scaffold 0 | 54,595 |
