# Supplementary material for: Sepsid even-skipped Enhancers Are Functionally Conserved in Drosophila Despite Lack of Sequence Conservation
Source: PLoS Genet. 2008 Jun 27;4(6):e1000106. doi: 10.1371/journal.pgen.1000106 (PMC2430619; doi:10.1371/journal.pgen.1000106)
Supplement: Table S3 — Pairwise sequence and binding site comparisons. (0.10 MB DOC) [file pgen.1000106.s007.doc]

Table S3. **Pairwise sequence and binding site comparisons**

| **Pairwise BLASTZ score (length x %ID)** | | | | | | | | | | | | | | | | |
| --- | --- | --- | --- | --- | --- | --- | --- | --- | --- | --- | --- | --- | --- | --- | --- | --- |
|  | ***D. mel*** | ***D. yak*** | ***D. ere*** | ***D. ana*** | ***D. per*** | ***D. pse*** | ***D. wil*** | ***D. vir*** | ***D. moj*** | ***D. gri*** | ***S. pun*** | ***S. cyn*** | ***D. sp*** | ***T. sup*** | ***T. min*** | ***T. put*** |
| *eve* stripe 2 | 700 | 585 | 700 | 282 | 160 | 189 | 151 | 143 | 113 | 140 | 60 | 61 | 59 | 86 | 90 | 59 |
| *eve* stripe 3+7 | 600 | 600 | 600 | 171 | 306 | 305 | 121 | 125 | 102 | 81 | 25 | 25 | 28 | 75 | 26 | 59 |
| *eve* stripe 4+6 | 800 | 800 | 800 | 284 | 398 | 404 | 232 | 288 | 224 | 0 | 0 | 0 | 0 | 0 | 0 | 31 |
| *eve* MHE | 612 | 612 | 612 | 238 | 212 | 213 | 60 | 171 | 152 | 0 | 0 | 0 | 0 | 0 | 37 | 0 |
| TOTAL |  | 2597 | 2712 | 976 | 1075 | 1111 | 564 | 727 | 590 | 221 | 85 | 86 | 87 | 160 | 152 | 148 |

| ***D. melanogaster* binding sites in BLASTZ hits** | | | | | | | | | | | | | | | | |
| --- | --- | --- | --- | --- | --- | --- | --- | --- | --- | --- | --- | --- | --- | --- | --- | --- |
|  | ***D. mel*** | ***D. yak*** | ***D. ere*** | ***D. ana*** | ***D. per*** | ***D. pse*** | ***D. wil*** | ***D. vir*** | ***D. moj*** | ***D. gri*** | ***S. pun*** | ***S. cyn*** | ***D. sp*** | ***T. sup*** | ***T. min*** | ***T. put*** |
| *eve* stripe 2 | 39 | 34 | 35 | 28 | 10 | 14 | 9 | 7 | 6 | 10 | 4 | 4 | 4 | 6 | 6 | 3 |
| *eve* stripe 3+7 | 49 | 45 | 47 | 21 | 34 | 34 | 16 | 17 | 18 | 16 | 3 | 3 | 3 | 17 | 3 | 8 |
| *eve* stripe 4+6 | 37 | 37 | 37 | 21 | 33 | 33 | 20 | 31 | 19 | 0 | 0 | 0 | 0 | 0 | 0 | 3 |
| *eve* MHE | 19 | 18 | 19 | 13 | 8 | 8 | 2 | 7 | 6 | 0 | 0 | 0 | 0 | 0 | 4 | 0 |
| TOTAL | 144 | 134 | 138 | 83 | 85 | 89 | 47 | 62 | 49 | 26 | 7 | 7 | 7 | 23 | 13 | 14 |

| **Conserved binding sites in BLASTZ hits** | | | | | | | | | | | | | | | | |
| --- | --- | --- | --- | --- | --- | --- | --- | --- | --- | --- | --- | --- | --- | --- | --- | --- |
|  | ***D. mel*** | ***D. yak*** | ***D. ere*** | ***D. ana*** | ***D. per*** | ***D. pse*** | ***D. wil*** | ***D. vir*** | ***D. moj*** | ***D. gri*** | ***S. pun*** | ***S. cyn*** | ***D. sp*** | ***T. sup*** | ***T. min*** | ***T. put*** |
| *eve* stripe 2 | 39 | 33 | 38 | 25 | 9 | 13 | 8 | 6 | 5 | 7 | 2 | 3 | 3 | 3 | 3 | 1 |
| *eve* stripe 3+7 | 49 | 35 | 42 | 17 | 23 | 23 | 15 | 15 | 12 | 21 | 1 | 1 | 3 | 9 | 2 | 4 |
| *eve* stripe 4+6 | 37 | 43 | 43 | 19 | 32 | 32 | 22 | 27 | 21 | 0 | 0 | 0 | 0 | 0 | 0 | 3 |
| *eve* MHE | 19 | 16 | 16 | 6 | 6 | 6 | 2 | 7 | 6 | 0 | 0 | 0 | 0 | 0 | 2 | 0 |
| TOTAL | 144 | 127 | 139 | 67 | 70 | 74 | 47 | 55 | 44 | 28 | 3 | 4 | 6 | 12 | 7 | 8 |

| **Conserved binding sites in multiple alignments** | | | | | | | | | | | | | | | | |
| --- | --- | --- | --- | --- | --- | --- | --- | --- | --- | --- | --- | --- | --- | --- | --- | --- |
|  | ***D. mel*** | ***D. yak*** | ***D. ere*** | ***D. ana*** | ***D. per*** | ***D. pse*** | ***D. wil*** | ***D. vir*** | ***D. moj*** | ***D. gri*** | ***S. pun*** | ***S. cyn*** | ***D. sp*** | ***T. sup*** | ***T. min*** | ***T. put*** |
| *eve* stripe 2 | 50 | 36 | 42 | 24 | 31 | 30 | 26 | 25 | 30 | 24 | 21 | 21 | 27 | 27 | 22 | 16 |
| *eve* stripe 3+7 | 40 | 35 | 28 | 25 | 15 | 27 | 20 | 26 | 27 | 24 | 9 | 10 | 12 | 13 | 12 | 10 |
| *eve* stripe 4+6 | 38 | 36 | 36 | 31 | 34 | 34 | 36 | 31 | 33 | 31 | 14 | 14 | 16 | 21 | 22 | 23 |
| *eve* MHE | 31 | 35 | 35 | 25 | 22 | 23 | 13 | 13 | 22 | 17 | 12 | 14 | 8 | 12 | 14 | 7 |
| TOTAL | 169 | 142 | 141 | 105 | 102 | 114 | 95 | 95 | 112 | 96 | 56 | 59 | 63 | 73 | 70 | 56 |
