# Supplementary material for: Sepsid even-skipped Enhancers Are Functionally Conserved in Drosophila Despite Lack of Sequence Conservation
Source: PLoS Genet. 2008 Jun 27;4(6):e1000106. doi: 10.1371/journal.pgen.1000106 (PMC2430619; doi:10.1371/journal.pgen.1000106)
Supplement: Table S4 — Predicted and tested sepsid enhancers. (0.09 MB DOC) [file pgen.1000106.s008.doc]

Table S4. **Predicted and tested sepsid enhancers**

| **Species** | **Enhancer** | **Source Sequence** | **Start** | **Stop** | **Length** | **Tested** |
| --- | --- | --- | --- | --- | --- | --- |
| *Sepsis cynipsea* | *eve* MHE | EU675304 | 19429 | 21144 | 1715 | yes |
| *Sepsis cynipsea* | *eve* stripe 2 | EU675304 | 7796 | 9734 | 1938 | yes |
| *Sepsis cynipsea* | *eve* stripe 3+7 | EU675304 | 4513 | 6556 | 2043 | yes |
| *Sepsis cynipsea* | *eve* stripe 4+6 | EU675304 | 17444 | 19447 | 2003 | yes |
| *Sepsis punctum* | *eve* MHE | EU675302 | 13024 | 14956 | 1935 |  |
| *Sepsis punctum* | *eve* stripe 2 | EU675302 | 733 | 2754 | 2021 |  |
| *Sepsis punctum* | *eve* stripe 3+7 | EU686389 | 23515 | 25634 | 2119 |  |
| *Sepsis punctum* | *eve* stripe 4+6 | EU675302 | 10943 | 13042 | 2099 |  |
| *Dicranosepsis sp.* | *eve* MHE | EU675301 | 24351 | 25853 | 1502 |  |
| *Dicranosepsis sp.* | *eve* stripe 2 | EU675301 | 9450 | 11881 | 2431 |  |
| *Dicranosepsis sp.* | *eve* stripe 3+7 | EU675301 | 6103 | 7642 | 1539 |  |
| *Dicranosepsis sp.* | *eve* stripe 4+6 | EU675301 | 20491 | 23842 | 3351 |  |
| *Themira putris* | *eve* MHE | EU675300 | 25604 | 27350 | 1746 |  |
| *Themira putris* | *eve* stripe 2 | EU675300 | 14657 | 16354 | 1697 | yes |
| *Themira putris* | *eve* stripe 3+7 | EU675300 | 11680 | 13361 | 1681 | yes |
| *Themira putris* | *eve* stripe 4+6 | EU675300 | 23554 | 25393 | 1839 | yes |
| *Themira superba* | *eve* MHE | EU675303 | 29063 | 30773 | 1710 | yes |
| *Themira superba* | *eve* stripe 2 | EU675303 | 18073 | 19863 | 1790 |  |
| *Themira superba* | *eve* stripe 3+7 | EU675303 | 15527 | 17413 | 1886 |  |
| *Themira superba* | *eve* stripe 4+6 | EU675303 | 26920 | 28864 | 1944 |  |
| *Themira minor* | *eve* MHE | EU675306 | 4959 | 6555 | 1596 |  |
| *Themira minor* | *eve* stripe 2 | EU675306 | 16151 | 17729 | 1578 |  |
| *Themira minor* | eve stripe 3+7 | EU675306 | 20677 | 22250 | 1573 |  |
| *Themira minor* | *eve* stripe 4+6 | EU675306 | 7621 | 9423 | 1802 |  |
