# Supplementary material for: Sepsid even-skipped Enhancers Are Functionally Conserved in Drosophila Despite Lack of Sequence Conservation
Source: PLoS Genet. 2008 Jun 27;4(6):e1000106. doi: 10.1371/journal.pgen.1000106 (PMC2430619; doi:10.1371/journal.pgen.1000106)
Supplement: Table S5 — Analyzed Drosophila enhancers. (0.12 MB DOC) [file pgen.1000106.s009.doc]

Table S5. **Analyzed *Drosophila* enhancers**

| **Species** | **Enhancer** | **Source Sequence** | **Start** | **Stop** | **Length** |
| --- | --- | --- | --- | --- | --- |
| *D. melanogaster* | *eve*_MHE | 2R | 5497096 | 5497708 | 612 |
| *D. melanogaster* | *eve*_stripe_2 | 2R | 5489500 | 5490200 | 700 |
| *D. melanogaster* | *eve*_stripe_3+7 | 2R | 5487300 | 5487900 | 600 |
| *D. melanogaster* | *eve*_stripe_4+6 | 2R | 5495711 | 5496511 | 800 |
| *D. simulans* | *eve*_MHE | CM000362.1 | 4503017 | 4503621 | 604 |
| *D. simulans* | *eve*_stripe_2 | CM000362.1 | 4497373 | 4498066 | 693 |
| *D. simulans* | *eve*_stripe_3+7 | CM000362.1 | 4493163 | 4493768 | 605 |
| *D. simulans* | *eve*_stripe_4+6 | CM000362.1 | 4501643 | 4502448 | 805 |
| *D. sechellia* | *eve*_MHE | CH480816.1 | 3486891 | 3487492 | 601 |
| *D. sechellia* | *eve*_stripe_2 | CH481150.1 | 11503 | 12195 | 692 |
| *D. sechellia* | *eve*_stripe_3+7 | CH481150.1 | 9205 | 9809 | 604 |
| *D. sechellia* | *eve*_stripe_4+6 | CH480816.1 | 3485511 | 3486322 | 811 |
| *D. yakuba* | *eve*_MHE | CM000157.2 | 18500783 | 18501397 | 614 |
| *D. yakuba* | *eve*_stripe_2 | CM000157.2 | 18492220 | 18492956 | 736 |
| *D. yakuba* | *eve*_stripe_3+7 | CM000157.2 | 18489908 | 18490564 | 656 |
| *D. yakuba* | *eve*_stripe_4+6 | CM000157.2 | 18499399 | 18500206 | 807 |
| *D. erecta* | *eve*_MHE | CH954177.1 | 8495654 | 8496299 | 645 |
| *D. erecta* | *eve*_stripe_2 | CH954177.1 | 8503243 | 8503997 | 754 |
| *D. erecta* | *eve*_stripe_3+7 | CH954177.1 | 8505487 | 8506139 | 652 |
| *D. erecta* | *eve*_stripe_4+6 | CH954177.1 | 8496893 | 8497758 | 865 |
| *D. ananassae* | *eve*_MHE | CH902619.1 | 15357368 | 15357982 | 614 |
| *D. ananassae* | *eve*_stripe_2 | CH902619.1 | 15364556 | 15365288 | 732 |
| *D. ananassae* | *eve*_stripe_3+7 | CH902619.1 | 15366311 | 15366847 | 536 |
| *D. ananassae* | *eve*_stripe_4+6 | CH902619.1 | 15358447 | 15359299 | 852 |
| *D. pseudoobscura* | *eve*_MHE | CM000071.2 | 10897570 | 10898200 | 630 |
| *D. pseudoobscura* | *eve*_stripe_2 | CM000071.2 | 10905809 | 10906752 | 943 |
| *D. pseudoobscura* | *eve*_stripe_3+7 | CM000071.2 | 10907927 | 10908572 | 645 |
| *D. pseudoobscura* | *eve*_stripe_4+6 | CM000071.2 | 10898690 | 10899714 | 1024 |
| *D. persimilis* | *eve*_MHE | CH479183.1 | 6220934 | 6221582 | 648 |
| *D. persimilis* | *eve*_stripe_2 | CH479183.1 | 6229296 | 6230190 | 894 |
| *D. persimilis* | *eve*_stripe_3+7 | CH479183.1 | 6231390 | 6232049 | 659 |
| *D. persimilis* | *eve*_stripe_4+6 | CH479183.1 | 6222072 | 6223098 | 1026 |
| *D. willistoni* | *eve*_MHE | CH963849.1 | 45059 | 45618 | 559 |
| *D. willistoni* | *eve*_stripe_2 | CH963849.1 | 33725 | 34347 | 622 |
| *D. willistoni* | *eve*_stripe_3+7 | CH963849.1 | 31366 | 31980 | 614 |
| *D. willistoni* | *eve*_stripe_4+6 | CH963849.1 | 38355 | 39270 | 915 |
| *D. mojavensis* | *eve*_MHE | CH933808.1 | 4438323 | 4439011 | 688 |
| *D. mojavensis* | *eve*_stripe_2 | CH933808.1 | 4429230 | 4430145 | 915 |
| *D. mojavensis* | *eve*_stripe_3+7 | CH933808.1 | 4419434 | 4420236 | 802 |
| *D. mojavensis* | *eve*_stripe_4+6 | CH933808.1 | 4438326 | 4439235 | 909 |
| *D. virilis* | *eve*_MHE | CH940648.1 | 1345584 | 1346066 | 482 |
| *D. virilis* | *eve*_stripe_2 | CH940648.1 | 1336890 | 1337748 | 858 |
| *D. virilis* | *eve*_stripe_3+7 | CH940648.1 | 1334576 | 1335429 | 853 |
| *D. virilis* | *eve*_stripe_4+6 | CH940648.1 | 1345374 | 1346611 | 1237 |
| *D. grimshawii* | *eve*_MHE | CH916367.1 | 9644981 | 9645882 | 901 |
| *D. grimshawii* | *eve*_stripe_2 | CH916367.1 | 9655560 | 9656447 | 887 |
| *D. grimshawii* | *eve*_stripe_3+7 | CH916367.1 | 9657479 | 9658456 | 977 |
| *D. grimshawii* | *eve*_stripe_4+6 | CH916367.1 | 9646350 | 9647842 | 1492 |
